# Using the H-index as a factor in the promotion of surgical faculty

**DOI:** 10.1016/j.heliyon.2022.e09319

**Published:** 2022-04-22

**Authors:** Rongzhi Wang, Marshall Lewis, Rui Zheng-Pywell, Janet Julson, Mary Smithson, Herbert Chen

**Affiliations:** Department of Surgery, University of Alabama at Birmingham, Birmingham, Alabama, United States

**Keywords:** Academic surgery, Academic productivity, H-index, Promotion

## Abstract

**Introduction:**

Academic productivity is an important determinant for promotion. However, the measurement of academic productivity is ill-defined. The aim of this study is to demonstrate the academic productivity at the time of promotions at our institution.

**Methods:**

We reviewed the data of 33 faculty from Department of Surgery at our institution who were promoted from 2006 to 2021. Gender, academic productivity at hiring, and each promotion were obtained. Academic productivity was assessed by bibliometric indices including total number of publications and citations, and H-index, which were obtained from Web of Science. T-test, Mann-Whitney U test, Fisher’s exact test and linear regression analysis were used to assess the association of H-index with length of promotion and gender. P < 0.050 were considered statistically significant.

**Results:**

The medians (interquartile ranges) of indexes at hiring, at promotions from assistant professors to associate professors, and from associate professors to full professors were 6.0 (1.5–9.5), 11.0 (9.0–18.0) and 17.0 (9.0–23.0) respectively. A simple linear regression showed significant correlation between the length of promotion to associate professors and their H-indexes at hiring. (F (1, 27) = 10.55, p = 0.003, R^2^ of 0.281.) There was no statistical significance in the difference of H-indexes at promotions between male and female faculty.

**Conclusion:**

At our institution, the median H-indexes at the time of promotions from assistant professor to associate professor and from associate professor to full professor are 11.0 and 17.0. Using the H-index as an objective measure can be a useful tool to junior surgical faculty as reference for applying promotion.

## 1 Introduction

Academic promotion is vital to both surgical faculty and departments of surgery. Promotion recognizes and awards the excellence of surgical faculty, which further accelerates the development of faculty’s careers and influence in academic surgery. Additionally, promotion creates a “culture of excellence” and helps departments attract new faculty members [1].

Historically, successful academic surgeons were expected to function equally as educators, scientists and clinicians. This so-called “triple-threat” concept was created a hundred years ago, by Sir William Osler, the Father of Modern Medicine and co-founding physician of Johns Hopkins Hospital [2]. Teaching, research, and clinical productivity are still considered important determinants for academic promotion along with other new tenets such as administrative duty and community service [3].

At our institution, faculty who prepare for promotion should provide curriculum vitae, recommendation reports/letters, evidence of teaching effectiveness, evidence of research productivity, summary of service activity, external reviewer letters and annual reviews [4]. Appointments to associate professor requires evidence of scholarly achievement in areas of research, teaching, and/or service, as appropriate, documented by peer recognition at a national level. Appointments to professor requires evidence of sustained scholarly achievement and productivity in the areas of research, teaching, and/or service and demonstration of nationally recognized excellence in the conduct of academic duties. Departments/divisions will review proposals and make decisions for approval or denial before March. Departments then submit all faculty promotion to Dean’s office. The School of Medicine Faculty Council reviews the promotion packet and send to department chair with recommendations for approval/denial of promotion. After hearing appeals for denied promotion, final decision is made in August [5].

Academic productivity is one way to measure research effectiveness in the process of promotion. While there is no golden standard metric to quantify academic productivity, markers including total number of publications as well as total number of publication citations are frequently used. In 2005, the Hirsch index (H-index) was introduced to measure both quantity and quality of research. The complicated equations were explicitly explained in the original publication [6] Given its consideration of both number of publications and number of citations for each publication, the H-index and number of publications are not in purely linear relation. The H-index is defined as h number of publications that have been cited at least h times [6] For example, for researchers who have an H-index of 7, they must have 7 publications with at least 7 citations each. The advantage of the H-index is that it provides a single number to indicate surgeons’ productivity and mitigates the skewed appearance of academic productivity that occurs when a small number of publications have a large number of citations, and vice-versa. Multiple studies have proven that academic productivity is associated with academic promotion [7, 8, 9, 10, 11, 12, 13]. It has been shown that the H-index offers better validity than other bibliometric indices of research performance in the surgical field [12, 13]. However, to our knowledge, literature identifying benchmarks of academic productivity requirement for promotion is lacking. An objective benchmark of quantified academic productivity would provide a blueprint for junior faculty members who are seeking academic promotion. Additionally, it functions as an evidence-based metric to evaluate candidates for promotions.

The purpose of this study was to retrospectively review the academic productivity of faculty promoted from assistant professors to associate professor and associate professor to full professor at our institution using H-index as a bibliometric measure.

## 2 Material and methods

### 2.1 Data collection

This was a retrospective study using data from the Department of Surgery at the University of Alabama at Birmingham (UAB) from 2006 to 2021. All the information obtained for the use in this study was obtained from publicly available information sources. Therefore, no institutional review board approval was required.

A total of 33 surgical faculty who were promoted from assistant professors to associate professors and/or associate professors to full professors in the period of 2006–2021 were identified. Most junior faculty were hired as assistant professors as this is the entry level position in our institution. Some faculty came to our institution as associate after practicing at other institutions. Bibliometric indices were obtained from Web of Science (https://www.webofscience.com) by searching authors' last names and first names. If similar author names existed in the database, categories and affiliations were used as cross-references. If the same author was listed under different authors’ records, all records were viewed as a combined record. Female faculty were searched by both maiden name and married name.

### 2.2 Study variables

Total number of publications, total times cited, H-index at the time of hiring, and promotions from assistant professors to associate professors and associate professors to full professors were collected to quantify research productivity. The length of promotion from assistant professors to associate professors and associate professors to full professors were calculated.

### 2.3 Statistical analyses

All descriptive statistics were tested for skewness. Continuous data with normal distribution was described as mean ± standard deviation. Continuous data with non-normal distribution was described as median and interquartile range (IQR). Normally distributed continuous data were compared using independent t-test and non-normally distributed continuous data were compared using the Mann-Whitney U test. Discrete variables were compared using Fisher’s exact test. Linear regression analysis was performed to the relationship between bibliometric indices and length of promotion from assistant professors to associate professors. IBM® SPSS® version 27.0 was used for data analyses. P < 0.050 was considered as statistically significant.

## 3 Results

Overall, 33 surgical faculty at our institution were promoted from 2006 to 2021. The majority of the faculty were male (73.5%), White (81.8%) and engaged in health service research (87.9%). This study is not powered to detect the differences between the group engaged in health service research and in basic science and the relationship between the type of research and H-index is beyond the scope of this study. There were 6 faculty specialized in gastrointestinal surgery, 5 in transplant surgery, 4 in pediatric surgery, 4 in surgical oncology, 4 in cardiothoracic surgery, 3 in trauma surgery, 3 in vascular surgery 2 in plastic surgery, 1 in breast surgery and 1 in endocrine surgery. There were 29 surgical faculty promoted from assistant professors to associate professors, 11 surgical faculty promoted from associate professors to full professors, and 7 surgical faculty promoted from assistant professors to associate professors and then associate professors to full professors. The award of tenure and promotion can be separate. In our study group, 8 out of 33 were not awarded tenure. Eight faculty were awarded tenure prior to promotion to association professor, 12 were awarded tenure at the time of promotion to association professor, and 5 faculty were awarded tenure after promotion to associate professor.

### 3.1 Productivity of surgical faculty

The academic productivity of surgical faculty at hiring, promotions from assistant professors to associate professors and associate professors to full professors are listed in Table 1.

**Table 1:** Productivity at hiring and promotions.

	Total publication	Total citations	H-index	Time for promotion (Years)
At hiring (n = 33)	10.0 (IQR 2.5–16.0)	180.0 (IQR 38.5–425.0)	6.0 (IQR 1.5–9.5)	-
At promotion from assistant professors to associate professors (n = 29)	28.0 (IQR 17.5–53.0)	503.0 (IQR 282.0–1217.5)	11.0 (IQR 9.0–18.0)	5.0 (IQR 4.5–7.0)
At promotion from associate professors to full professors (n = 11)	41.0 (IQR 24.0–73.0)	1132.0 (IQR 441.0–1321.0)	17.0 (IQR 9.0–23.0)	6.0 (IQR 5.0–9.0)

At hiring, faculty had a median number of 10.0 (IQR 2.5, 16.0) publications, median number of 180.0 (IQR 38.5, 425.0) citations, and median H-index of 6.0 (IQR 1.5, 9.5).

At promotion from assistant professors to associate professors, faculty had a median number of 28.0 (IQR 17.5, 53.0) publications, median number of 503.0 (IQR 282.0, 1217.5) citations, and median H-index of 11.0 (IQR 9.0, 18.0). The median time required for promotion from assistant professors to associate professors was 5.0 (IQR 4.5, 7.0) years.

At promotion from associate professors to full professors, faculty had a median number of 41.0 (IQR 24.0, 73.0) publications, median number of 1132.0 (IQR 441.0, 1321.0) citations, and median H-index of 17.0 (9.0, 23.0). It took a median time of 6.0 (IQR 5.0, 9.0) years for surgical faculty in our institution to be promoted from associate professors to full professors.

### 3.2 Productivity versus length of promotion from assistant professors to associate professors

Based on the median length of time required for promotion from assistant professors to associate professors, promoted faculty were divided into two groups: promotion length ≤5 years and promotion length >5 years. The academic productivity of the two groups are listed in Table 2. Surgical faculty who were promoted to associate professors in ≤5 years had more publications at hiring (12 (IQR 7, 26) versus 6.5 (IQR 1.8, 10.0) p = 0.003) and higher H-index scores at hiring (8.0 (IQR 5.0, 12.0) versus 4.5 (IQR 1.0, 8.0) p = 0.020). There were no differences in number of total citations at hiring, total publications at promotion, total citations at promotion or H-index at promotion between two groups.

**Table 2:** Productivity versus Promotion length from assistant professors to associate professors.

	Promotion length≤5 years (n = 15)	Promotion length>5 years (n = 14)	Mann-Whitney U test
Total publication at hiring	12 (IQR 7–26)	6.5 (IQR 1.8–10.0)	P = 0.003∗
Total citation at hiring	258.0 (IQR 172.0–1486.0)	155.0 (IQR 16.5–350.8)	P = 0.134
H-index at hiring	8.0 (IQR 5.0–12.0)	4.5 (IQR 1.0–8.0)	P = 0.020∗
Total publication at promotion	38.0 (IQR 17.0–68.0)	27.5 (IQR 16.5–42.0)	P = 0.400
Total citation at promotion	503.0 (IQR 273.0–1487.0)	501.5 (IQR 282.0–1047.0)	P = 0.983
H-index at promotion	11.0 (IQR 9.0–20.0)	11.5 (IQR 8.25–18.0)	P = 0.983

∗ Indicates statistically significant.

A simple linear regression was calculated for length of promotion to associate professors based on their H-index at hiring. A significant regression equation was found (F (1, 27) = 10.55, p = 0.003), with an R^2^ of 0.281. When the H index at hiring is zero, the mean length of promotion to associate professor is 7.0 years. Based on these findings, 28.1% of the time to promotion to associate professor is explained by the H-index at hiring. Specifically, as the H-index at hiring increases by one point, there is an associated mean decrease in time to of promotion by 0.17 years.

### 3.3 Gender versus promotion

There were 7 female faculty versus 22 male faculty who were promoted from assistant professors to associate professors (Table 3). Female faculty tended to have more publications, more citations, and higher H-index at hiring and promotions than male faculty, however, these findings were not statistically significant.

**Table 3:** Gender versus productivity at promotions to associate professors.

	Female (n = 7)	Male (n = 22)	
Total publication at hiring	11.0 (IQR 4.0–16.0)	9.5 (IQR 2.8–17.8)	P = 0.901
Total citation at hiring	195.0 (IQR 32.0–324.0)	181.5 (IQR 82.5–491.0)	P = 0.940
H-index at hiring	7.0 (IQR 2.0–10.0)	6.5 (IQR 2.5–9.5)	P = 0.823
Total publication at promotion	31.0 (IQR 18.0–51.0)	27.5 (IQR 16.8–55.8)	P = 0.784
Total citation at promotion	511.0 (IQR 253.0–869.0)	497.5 (IQR 282.0–1510.5)	P = 0.940
H-index at promotion	12.0 (IQR 9.0–18.0)	11.0 (IQR 8.8–18.5)	P = 0.940
Length of promotion to associate professors	6.0 ± 1.6	5.6 ± 2.5	P = 0.719

There were 3 female faculty versus 8 male faculty who were promoted from associate professors to full professors (Table 4). Again, it is noticed that female faculty tended to have more research productivity than their male counterpart, however, the differences were not statistically significant.

**Table 4:** Gender versus productivity at promotions to full professors.

	Female (n = 3)	Male (n = 8)	
Total publication at promotion	65.3 ± 33.2	38.6 ± 23.3	P = 0.161
Total citation at promotion	1220.0 (IQR 346.0, -)	885.0 (IQR 482.8–1730.5)	P = 0.776
H-index at promotion	16.7 ± 7.8	15.5 ± 7.3	P = 0.822
Length of promotion to full professors	5.0 (IQR 5.0, -)	6.0 (IQR 5.0–8.5)	P = 1.000

## 4 Discussion

Getting promoted in academia is a measure of success, recognition of endeavor, and motivation to achieve excellence for surgical faculty. From medical school to fellowship application, objective benchmarks are constantly available for public reference such as Medical College Admission Test (MCAT), United States Medical Licensing Examination (USMLE) for residency, and American Board of Surgery In-Training Exam (ABSITE). However, the process of promotion in academic surgery is less clear and lacks objective measurement. The decisions made by medical school or residency/fellowship program to accept an applicant is multifactorial just like promotion and therefore, objective benchmarks are not the only tool used for making the final decision but rather can serve as a reference to help standardize the applicants. In this study, we aimed to find an objective measurement of scholarly activities for promotion.

Traditionally, total number of publications and citations were used to measure academic achievement. However, results can be skewed by individuals with large number of low impact publications or very few publications but with high number of citations. The introduction of H-index in 2005 mitigated this bias. Since then, H-index has been widely adopted to measure impact in academic surgery. In previous studies, higher H-indexes are consistently associated with higher academic ranks across different surgical specialties [8, 9, 10, 11, 14, 15, 16, 17, 18, 19] (see Table 5). The H-indexes of assistant professors, associate professors and full professors ranged from 2 to 11.5, 10 to 17 and 17–48 respectively. The large range of H-indexes can partly be explained by the differences in surgical specialties and subspecialties. In our study, there is a large range of H-indexes at promotions in different surgical subspecialties. This study is not powered to detect the difference of H-indexes among different subspecialties, however, we did find faculty in certain subspecialties had higher H-indexes. For example, at the promotion from assistant professors to associate professors, faculty in breast surgery have the lowest median of H-index (8.0) and faculty in transplant surgery have the highest median of H-index (29.0). This echoed the findings of differences of H-indexes in other studies. LaRocca et al. (2018) [11] and Lafaro et al. (2020) [10] reported higher H-indexes across three academic ranks in surgical oncology and hepatopancreatobiliary surgery compared to other surgical specialties. Ashfaq et al (2018) showed similar findings and reported higher H-indexes in thoracic surgeons and surgical oncologists. The competitive nature of these specialties likely additional research time to match into fellowship [14].

**Table 5:** H-index by surgical specialty and academic ranks.

Author	Year	Specialty	Number of faculty in the study	H-index (Assistant Professor)	H-index (Associate Professor)	H-index (Full Professor)
Ashfaq et al. [14]	2018	General surgery	3712	Mean ± SD 6.8 ± 64	12.9 ± 9.3	27.9 ± 17.4
Lopez et al. [15]	2015	Hand Surgery	366	Mean ± SD 5 ± 4	10 ± 7	18 ± 9
Radford et al. [16]	2021	Breast Surgery	209	Median (Range) 6 (1–37)	17 (4–48)	37 (6–114)
Ence et al. [8]	2016	Orthopedic Surgery	4663	Median (IQR) 2 (1–6)	8 (3–14)	17 (10–27)
Gast et al. [9]	2014	Plastic Surgery	127	Mean ± SD 8.2 ± 5.8	15.4 ± 6.9	25.0 ± 13.5
LaRocca et al. [11]	2018	Surgical Oncology	319	Median (Range) 7 (1–30)	17 (3–44)	39 (6–138)
Desai et al. [17]	2018	Pediatric Surgery	430	Mean ± SD 10.35 ± 6.60	14.17 ± 6.45	25.55 ± 11.04
Lafaro et al.	2020	Hepatopancreatobiliary Surgery	111	Median (Range) 11.5 (0–26)	22.0 (3–40)	48.0 (12–97)
Han et al. [18]	2018	Oral and Maxillofacial Surgery	299	Mean ± SD 3.9 ± 3.5	7.7 ± 5.4	15.5 ± 10.4
Tomei et al. [19]	2014	Neurological Surgery	1052	Median (IQR) Male: 6 (3–10) Female: 4 (2–9)	Male: 10 (6–16) Female: 9 (6–15)	Male: 17 (10–27) Female: 15 (12–23)

In our study, we found that the median (IQR) of indexes of surgery faculties at hiring, at promotion from assistant professors to associate professors, and at promotion from associate professors to full professors were 6.0 (1.5–9.5), 11.0 (9.0–18.0), and 17.0 (9.0–23.0). The H-indexes from our study were relatively lower compared to most of the other studies. This finding can be explained by the timing when the data was collected. Our study captured H-indexes in the year of promotions, however, all other studies were snapshots of the year when the studies were performed. The H-indexes increase as time progresses after promotion. Geographic regions may also be a contributing factors. Svider et al. (2013) [20] and Ence et al. (2016) [8] in their studies found that academic faculty within otolaryngology and orthopedic surgery had significantly lower H-indexes in southern region comparing to other geographic regions in.

It has been reported in multiple studies that female faculty are underrepresented compared to their male counterparts in different surgical specialties [16, 21, 22, 23, 24, 25, 26, 27, 28] Female faculty reportedly have overall lower H-indexes compared to male faculty in academic surgery [23, 25, 29, 30, 31]. However, when stratified by academic rank, previous studies showed different results of gender disparities of H-index at each academic rank. A study by Gawad et al. found that male faculty compared to female have higher H-index at assistant professor rank with no differences at associated professor rank and full professor rank. (2020) [21], which was similar to the findings of the studies of general surgeons in Canada from Mueller et al (2016) [32] and Orthopedic surgeons in the U.S. from Hoof et al (2020) [24]. On the contrary, Nguyen et al. (2018) [30] showed that male faculty had higher H-indexes than female faculty across all ranks in surgical oncology. When stratified by years in academia, Mayer et al. (2017) found no differences in academic productivity between different genders across all academic ranks in urology. Carnevale et al. (2020) [25] found that female faculty had higher average H-index per year than male faculty in vascular surgery. Several factors could explain the discrepancies in H-index when comparing genders including length of practice as well as other factors of the academic promotion process including teaching and clinical expertise. By capturing the H-indexes of surgical faculty at promotion, we reduced the confounding factor of time since starting practice in our study. We did not identify a difference in H-index at time of hiring or promotion between male and female faculty within our study. (Tables 3 and 4). Based on our findings, we believe that H-index serves as a suitable measure of academic productivity for both male and female faculty applying for academic promotion.

There are limitations to the use of H-index as a measure of scholarly effectiveness in the process of promotion evaluation. One concern is that H-index remains the same or is most likely to increase over time as surgical faculty who stay in academia longer. Even if the faculty do not publish any new papers, their total number of publications will remain the same and the number of citations of each paper will either remain the same or increase. The increasing number of citations over time will cause the H-index to grow even if the number of publications remains the same. Additionally, the H-index does not take into account orders of authorship despite the fact that first authors and senior authors usually have more academic input than second authors. Another limitation is that some citations are used with no real significance to the publication or may even be used in a negative context. Other confounding factors such as the “Matthew effect”, whereby well-stablished researchers and projects are cited more often than those that are less known [33]. Furthermore, there are different bibliometric search engines available and the results of H-index can vary in different search engines. We chose Web of Science, which has a more extensive coverage of publications and their citations from 1990 to present. In our study, 28.1% of the length of promotion to associate professor is explained by the H-index at hiring. This indicates that the evaluation of academic promotion is affected by multiple factors besides H-index such as teaching, clinical productivity and other aspects of academic productivity such as grant funding.

To our knowledge, this is the first study examining H-index as an objective measurement of academic productivity by capturing bibliometric index at the time of promotions. As we mentioned, the process of evaluating academic promotion involves different routes and multiple aspects. The study results showed that H-index was not perfect indicator of academic promotion. However, it still provides junior surgical faculty a useful reference for academic promotion.

## 5 Conclusion

There are multiple components to achieving promotion in the field of academic surgery. Currently, an objective standard to measure academic productivity as a determinant of promotion is lacking. At our institution, the median H-indexes at the time of promotions from assistant professor to associate professor and from associate professor to full professor are 11.0 and 17.0. Using the H-index as an objective measure can be a useful tool to junior surgical faculty as a reference for promotion application.

## Declarations

### Author contribution statement

Rongzhi Wang: Performed the experiments; Analyzed and interpreted the data; Wrote the paper.

Marshall Lewis: Performed the experiments; Analyzed and interpreted the data.

Rui Zheng-Pywell, Janet Julson, Mary Smithson: Analyzed and interpreted the data.

Herbert Chen: Conceived and designed the experiments.

### Funding statement

This research did not receive any specific grant from funding agencies in the public, commercial, or not-for-profit sectors.

### Data availability statement

Data will be made available on request.

### Declaration of interests statement

The authors declare no conflict of interest.

### Additional information

No additional information is available for this paper.
